# Determination of residues of pesticides, anabolic steroids, antibiotics, and antibacterial compounds in meat products in Oman by liquid chromatography/mass spectrometry and enzyme-linked immunosorbent assay

**DOI:** 10.14202/vetworld.2021.709-720

**Published:** 2021-03-22

**Authors:** Issa Al-Amri, Isam T. Kadim, Abdulaziz AlKindi, Ahmed Hamaed, Rabea Al-Magbali, Samera Khalaf, Khdija Al-Hosni, Fazal Mabood

**Affiliations:** 1 Department of Biological Sciences and Chemistry, College of Arts and Sciences, University of Nizwa, PO Box 33, PC 616, Birkat Al-Mouz, Nizwa, Sultanate of Oman; 2 Department of Animal and Veterinary Sciences, College of Agriculture and Marine Sciences, Sultan Qaboos University, Muscat, Sultanate of Oman; 3 Natural and Medical Sciences Research Center, University of Nizwa, PO Box 33, PC 616, Birkat Al-Mouz, Nizwa, Sultanate of Oman; 4 Institute of Chemical Sciences, University of Swat, Khyber Pakhtunkhwa, Pakistan

**Keywords:** anabolic steroids, antibiotic, enzyme-linked immunosorbent assay, liquid chromatography/mass spectrometry, meat products, pesticides, residue

## Abstract

**Background and Aim:**

Meat is a rich source of many nutrients and plays a vital role in human life however, meat safety is one of the top priorities of great concern for consumers today. More than 90% of human exposure to harmful materials is due to consumption of contaminated meat products. This study was designed to compare four valid analytical methods for the determination of organochlorine pesticides 2,4 D (2,4-dichlorophenoxyacetic acid), dichlorodiphenyldichloroethylene/dichlorodiphenyltrichloroethane, alachlor, organophosphate, anabolic steroids (progesterone, testosterone, and estrogen), antibiotics (tetracycline, sulfonamides, gentamycin, and cephalexin), antibacterial compounds (Macrolide, ß-Lactam, Chloramphenicol, Sulphur drugs, and Gentamicin) residues in 135 beef, buffalo, and sheep meat samples (fresh, frozen meats, minced, and sausage samples) of local, regional, and international brands available in Omani markets.

**Materials and Methods:**

Triplicate meat samples from each brand within each species were extracted with acetonitrile and purified with acetonitrile-saturated n-hexane to remove all impurities. To dry the sample after heating, the residue was passed across a Sep-Pak C18 cartridge for sample cleaning before gas chromatography (GC) (Brand GCMS-QP2010 Plus) coupled with different detectors, including a mass spectrometer or GC-electron capture detector (GC-ECD). Liquid chromatography/mass spectrometry (LC-MS) was also employed for the quantification of the residues in meat products. Enzyme-linked immunosorbent assay (ELISA) kits were employed to assess veterinary drug residues, anabolic steroids, and pesticides. The CHARM II instrument was employed to detect chloramphenicol, gentamicin, sulfa-drug, ß-lactam, and macrolide residues in meat and meat product samples.

**Results:**

A thin-layer chromatographic (TLC) method should be considered as another method of choice to determine concentrations of veterinary drugs and anabolic steroids. The TLC results were validated by LC-MS. The three described methods permit the multi-residue analysis of anabolic steroid residue levels of 0.06-1.89 ppb in meat product samples. There were three violative residues of anabolic steroids in red meat products that were above the maximum residue limits (MRLs). Although, the levels of organochlorine pesticides and antibiotic concentrations in meat products were below the MRLs, the long-term consumption is considered a health hazard and will affect the wellbeing of consumers.

**Conclusion:**

The four techniques (GC, high-performance liquid chromatography, ELISA and CHARM II) provided results that were reliable and precise for the detection of chessssmical residues in meat and meat products.

## Introduction

It has been forecasted that the global population will increase to nine billion by 2050 [1]. The increase in population will lead to an increase in the demand for animal proteins. Meat products are important ingredients in the human diet due to their richness in proteins, essential amino acids, and vitamins [2,3]. Although the consumption of meat products is increasing, consumers are concerned about the food safety of the meat products currently available. However, animal production is continuously developing and residues of veterinary drugs, anabolic steroids and pesticides have become an important issue for meat product safety [4-7]. Chemical residues in meat products may potentially be harmful to human health, so the healthy image of meat products can be influenced by the presence of residues [8-11]. Some harmful residues in meat products may come from feed and water consumed [12,13]. Pesticides including 2,4D, dichlorodiphenyldichloroethylene (DDE)/dichlorodiphenyltrichloroethane (DDT), tetrachlorodibenzodioxin (TCDD), triclosan, alachlor, and organophosphate carbonate are ubiquitous in Oman and can lead to serious public health hazards such as hormonal disruptions, immune system disturbances, as well as cancer [5,14]. Residues of many types of pesticides have been detected in meat products [5,15]. According to Bantobal and Jodral [16], more than 90% of consumer’s exposure to dangerous chemical residues is attributed to the consumption of contaminated animal products, including meat. As antimicrobial drugs, antibiotics are also widely prescribed for animals and their residues have been detected in animal tissues [10,17-21].

The Sultanate of Oman is a public market for numerous types of meat products from all over the globe. To assess the chemical hazard levels due to the accumulation of the various contaminants in meat products the maximum residue limits (MRLs) have been used. Residues in meat and meat products at certain levels may be harmful to Omani consumers [22]. Although, harmful levels of pesticide, antimicrobial and anabolic steroid residues in animal products are rare, a low incident remains a principal public health concern [23]. It has been stated that even low contaminant residues in meat products with long-term exposure might cause serious human health hazards in Oman and elsewhere [15,24,25].

Because of increasing public awareness of the safety of meat products in Oman, the need for sensitive, selective and dependable analytical methods to detect, monitor, and quantify chemical contaminants is more important than ever before. This study was aimed to employfour different techniques to determine the levels of several pesticides, anabolic steroids, antibiotics, and antibacterial compounds in 135 meat samples (cattle, buffalo, and sheep) representing, fresh, frozen, minced, burger, and sausages available in Omani markets.

## Materials and Methods

### Ethical approval

This investigation did not use live animals; therefore, ethical approval was not necessary.

### Study location and period

This study was carried out between September 2017 to June 2020 at Muscat region, Sultanate of Oman. The samples were processed at the Department of Animal and Veterinary Sciences, College of Agricultural and Marine Sciences, Sultan Qaboos University, Muscat, Sultanate of Oman.

### Meat samples

One-hundred and thirty-five meat samples (five replicates) representing three species of beef, sheep and buffalo, and four meat products (fresh: Four brands, frozen: Eight brands, minced: 12 brands, and sausage: Three brands) and different brands were randomly collected from five different food supply stores in Oman represented different meat-producing animal species from local markets in Nizwa, Oman (Table-1). The meat samples were kept in zipped plastic bags, transported in an insulated icebox, and stored at −18°C until analysis.

**Table-1:** The brand name of red meat products (five replicates for each brand; 27 brands×5 = 135 samples) collected from local market.

Beef					Sheep		Buffalo	
Fresh	Frozen	Minced	Sausage	Fresh	Frozen	Minced	Frozen	Minced
Brand 1	Brand 1	Brand 1	Brand 1	Brand 1	Brand 1	Brand 1	Brand 1	Brand 1
Brand 2	Brand 2	Brand 2	Brand 2	Brand 2		Brand 2	Brand 2	
		Brand 3	Brand 3			Brand 3	Brand 3	
		Brand 4				Brand 4	Brand 4	
		Brand 5				Brand 5	Brand 5	
		Brand 6						

### Reagents

Individual stock solutions of organochlorine pesticides standards of carbadox 50 mg and penicillin, cephalexin, aminoglycoside, tetracycline, and sulfanilamide (100 mg of each) with purities of >99% were prepared in acetonitrile, whereas (Clopidol: CLP 100 mg) was prepared in acetonitrile/water (1/1, v/v) solution. Working standard solutions for each antibiotic were diluted with acetonitrile (0.05 M) and sodium hydrogen phosphate (3/7, v/v) to a series of concentrations ranging from 0.2 to 2.0 mg/mL.

### Sample extraction and cleanup

A multitude of techniques is available for sample preparation depending on the character of the sample, the matrix, and the target residue [26]. The methods used for extraction of multi-residues (pesticides, antibiotic, anabolic steroids, and antibacterial) were carried out according to Minkao *et al*. [27]. The concentration of pesticide residues in red meat samples was obtained by gas chromatography/mass spectrometer (GC/MS) (Shimadzu-Japan GC-MS system QP2010 Ultra with GC-2010 plus) advanced flow controller. However, the concentration of antibiotics was determined using thin-layer chromatography (TLC) and validated by liquid chromatography-tandem mass spectrometry (LCMS/MS) (HLPC-Ultimate 3000, Dionex Softron GmbH, Dormiersir 4 D8821, Germany) following the procedure described by Tajick and Shohreh [28]. Residues of chloramphenicol, gentamicin, sulfa-drug, ß-Lactam, and Macrolide contaminants were detected using CHARM II. The high-performance liquid chromatography (HPLC) was also used to detect residues of the veterinary drugs, and alachlor, TCDD, DDT, 2,4-dichlorophenoxyaceticacid, organophosphate, tridosan, and carbamate (OP/C), and pesticides in meat and meat products. Antimicrobial residues and contaminants with anabolic activity can be tested by cost-effective and rapid immunochemical techniques, including radioimmunoassays, enzyme-linked immunosorbent assays (ELISA) or microbial growth inhibition assays [29]. Therefore, ELISA (ELISA: Multican Go-Type 1510. Thermo-Fisher Scientific OY, Finland) kits were used to determine antibiotics (streptomycin, tetracycline, sulfamethazine, and sulfamethoxazole), hormones (progesterone, testosterone, trenbolone, and estrogen), and pesticides (2,4D, DDE/DDT, TCDD, triclosan, alachlor, and organophosphate carbonate) following the procedure described by Ibrahim *et al*. [15]. The CHARM II instrument was used to detect chloramphenicol, gentamicin, sulfa-drug, ß-lactam, and macrolide residues in red meat and meat products from the local market. A general procedure was used to detect five different classes of antibacterial residues in meat products.

### Pesticide

Approximately 5 g meat sample from each brand was homogenized with 20 mL acetonitrile in a 50 mL centrifuge tube usingUltra Turrax T25 homogenizer (IKA Works Inc., USA). Acetonitrile (20 mL) was added and shaken for 3 min, then the mixture was filtered and the residues mixed with 50 mL of acetonitrile. The filtrated mixtures were moved into a separation funnel containing 30 mL of acetonitrile-saturated n-hexane and shaken for 5 min. The acetonitrile layer was gathered and evaporated to dryness at 40°C using a rotary evaporator, and then was mixed with 20 mL of (0.05 M NaH_2_PO_4_) and introduced in a Sep-Pak C18 cartridge. The concentration was washed twice with 5 mL of NaH_2_PO_4_ and then applied to the cartridge. The eluate was discarded and the flask was washed twice with 5 mL of methanol and the solution was passed through the cartridge. The eluate was evaporated to dryness at 40°C and then reconstituted with 1 mL of acetonitrile/water (3/7, v/v) solution. After spiking 0.5 mL of acetonitrile-saturated n-hexane, the solution was thoroughly mixed and then centrifuged at 3000 rpm for 5 min. The acetonitrile layer was collected and filtered through a membrane filter of 45 mm into vial and stored in a freezer at −80°C until analysis.

The temperature of GC/MS analysis of pesticides was set at 275°C in spilt less mode with a 10.6 psi pressure constant flow. The flow of He through a GC column was set at 1 mL/min. The oven program is: 100°C for one min, ramp at 20°C column was set at 140°C, then ramp at 5°C/min until reached 280°C, and then held for 8 min. Interface temperature of the GC to the MS was set at 250°C and the MS ion source was set at 200°C. The MS was operated in electron–ionization mode scan ranged 60-500 m/z. The GC/MS was calibrated with each new sample batch. The calibration range for GC/MS is 200-1000 g/L.

### Antibiotics and anabolic steroids

TLC was used to detect antibiotic and anabolic steroid residues by following the procedure of Tajick and Shohreh [28]. Two grams of meat sample from each brand were homogenized with 5 mL phosphate buffer (pH 6.5). The protein was precipitated by adding 1 mL of trichloro-acetic acid (30%). The solvent was transferred to a 15 mL centrifuge tubes and centrifuged at 7000 rpm for 15 min. The supernatant was collected, and then extracted by an equal volume of diethyl ether. The mixture was stored atroom temperature (23-25ºC) for 10 min and then separated using a separating funnel. The upper oily layer was discarded and the bottom layer was collected. The steps were repeated 5-8 times with diethyl ether and evaporated until dryness. The evaporated sample was reconstituted with 2 mL of mobile phase (methanol and acetone 1:1) and kept in a refrigerator for analysis of antibiotics and anabolic steroids.

After washing the glass plates (10×20 cm dimensions) in an acetone bath, 2 g of silica gel F256 (Merck, Germany) mixed in 5 mL distilled water in each plate and shaken thoroughly to create a fine paste. Each glass plate was coated with 0.25 mm thickness of silica paste by a TLC gel spreader system (Shandon, England). Plates were activated at 120°C for 2 h. Raw antibiotics were prepared by dissolving 0.1 g of each material in 4 mL methanol [30]. Approximately 50 mL of methanol dissolved antibiotic were applied at certain point on the line of the silica plates. The treated sample was moved to TLC tank containing acetone-methanol (1:1) as mobile phase. After the solvent front reaching to end of plates, chromatograms observed on ultraviolet light at 256 nm [30].

### Antibacterial compounds

The Charm II system (LSC 7600 System: Liquid Scintillation Counter/Illuminometer, USA) offers widespread food safety testing for numerous food media. It has been used to test a variety of chemical residues in foods, including meat products [9]. In the current study, the Charm II instrument was used to detect chloramphenicol, gentamicin, sulfa-drug, beta-lactam, marcrolide, amphenicol, streptomycin, and tetracycline residues in meat products available in Omani markets. A general procedure similar to that used for other residues was used to detect eight residues of antibacterial compounds in meat and meat products in duplicate.

## Results and Discussion

The results for the 135 red meat product samples analyzed are presented in Table-2 for fresh beef meat, Table-3 for fresh sheep meat, Table-4 for frozen beef and sheep meat, Table-5 for frozen buffalo meat, Table-6 for beef and buffalo mincemeat, Table-7 for sheep mincemeat, and Table-8 for beef sausage, Overall, the results obtained by the HPLC and the ELISA methods were in a similar range for most of the tested samples.

**Table-2:** Mean concentrations of residues of pesticides, anabolic steroids, antibiotics, and antibacterial compounds (ng/g) in 5 samples of fresh beef meat from markets in Oman.

Compound (μg/g)	Brand 1			Brand 2		
	
	HPLC^1^/GC^3^	CHARM	ELISA^2^	HPLC/GC	CHARM	ELISA
Pesticides						
2,4 D	1.11	-----	14.5	16.2	-----	17.4
DDE/DDT	22.1	-----	28.9	31.9	-----	33.2
Alachlor	0.11	-----	0.14	0.00	-----	0.00
Organophosphate	1.65	-----	1.78	2.22	-----	1.99
Anabolic steroids						
Testosterone	0.00	-----	0.00	0.00	-----	0.00
Trenbolone	0.36	-----	0.95	0.93	-----	0.87
Estrogen	2.19	-----	1.24	1.32	-----	0.96
Antibiotics						
Streptomycin	3.21	-----	1.88	0.00	-----	1.95
Tetracycline	0.90	-----	0.89	1.51	-----	0.88
Sulfamethazine	0.36	-----	0.33	0.00	-----	0.39
Sulfamethoxazole	1.09	-----	1.15	0.00	-----	1.24
Antibacterial compounds						
Triclosan	0.29	-----	0.34	0.00	-----	0.20
Amphenicol	-----	0.00	-----	-----	0.00	-----
Macrolide	-----	0.00	-----	-----	0.00	-----
ß-Lactam	-----	0.00	-----	-----	0.00	-----
Chloramphenicol	-----	0.00	-----	-----	0.00	-----
Gentamicin	-----	0.00	-----	-----	0.00	-----

1 HPLC=High-performance liquid chromatography.

2 ELISA=Enzyme linked immunosorbent assay. Pesticides were determined by

3 GC=Gas chromatograph-mass spectrometer. Maximum residue limits (ng/g)=MRL streptomycin (500), tetracycline (100), sulfamethazine (100), sulfamethoxazole (100), triclosan (10), testosterone (0.1), trenbolone (10), estrogen (0.1), 2,4 D (50), DDE/DDT (1000), alachlor (100). DDE=Dichlorodiphenyldichloroethylene, DDT=Dichlorodiphenyltrichloroethane

**Table-3:** Mean concentrations of residues of pesticides, anabolic steroids, antibiotics, and antibacterial compounds (ng/g) in five samples of fresh sheep meat samples available in local markets in Oman.

Chemical contaminants	Brand 1			Brand 2		
	
	HPLC^1^/GC^2^	CHARM	ELISA^3^	HPLC/GC	CHARM	ELISA
Pesticides						
2,4 D	22.8	-----	26.3	13.4	-----	14.2
DDE/DDT	-----	-----	-----	-----	-----	-----
Alachlor	1.11	-----	1.14	0.49	-----	0.55
Organophosphate	-----	-----	-----	-----	-----	-----
Anabolic steroids						
Testosterone	0.06	-----	0.05	0.07	-----	0.02
Trenbolone	0.13	-----	0.68	0.74	-----	0.51
Estrogen	1.38	-----	1.26	1.42	-----	1.33
Antibiotics						
Streptomycin	0.00	-----	0.00	0.00	-----	0.00
Tetracycline	0.41	-----	0.51	4.21	-----	3.99
Sulfamethazine	0.00	-----	0.00	0.00	-----	0.00
Sulfamethoxazole	1.21	-----	1.13	1.09	-----	1.13
Antibacterial compounds						
Triclosan	0.29	-----	0.35	1.00	-----	0.95
Amphenicol	-----	-----	-----	-----	-----	-----
Macrolide	-----	-----	-----	-----	-----	-----
ß-Lactam	-----	-----	-----	-----	-----	-----
Chloramphenicol	-----	-----	-----	-----	-----	-----
Gentamicin	-----	-----	-----	-----	-----	-----

1 HPLC=High-performance liquid chromatography. Pesticides were determined by

2 GC=Gas chromatography-mass spectrometry.

3 ELISA=Enzyme linked immunosorbent assay. Maximum residue limits (ng/g)=MRL streptomycin (500), tetracycline (100), sulfamethazine (100), sulfamethoxazole (100), triclosan (10), testosterone (0.1), trenbolone (10), estrogen (0.1), 2,4 D (50), DDE/DDT (1000), Alachlor (100). DDE=Dichlorodiphenyldichloroethylene, DDT=Dichlorodiphenyltrichloroethane

**Table-4:** Mean concentrations of residues of pesticides, anabolic steroids, antibiotics, and antibacterial compounds (ng/g) in five samples of frozen beef and sheep meat samples available in local markets in Oman.

Contaminant	Beef						Sheep		
	
	Brand 1			Brand 2			Brand 1		
		
	HPLC^1^/GC^3^	CHARM	ELISA^2^	HPLC/GC	CHARM	ELISA	HPLC/GC	CHARM	ELISA
Pesticides									
2,4 D	0.00	-----	32.2	-----	-----	31.0	29.9	-----	30.7
DDE/DDT	0.00	-----	0.00	-----	-----	0.00	0.00	-----	0.00
Alachlor	0.00	-----	1.08	-----	-----	1.72	0.51	-----	0.48
Organophosphate	0.00	-----	1.84	-----	-----	1.92	2.22	-----	1.88
Anabolic steroids									
Testosterone	1.24	-----	1.33	1.16	-----	1,11	0.00	-----	0.00
Trenbolone	1.19	-----	1.19	1.12	-----	0.63	0.53	-----	0.29
Estrogen	1.82	-----	1.82	1.65	-----	1.77	1.52	-----	0.54
Antibiotics									
Streptomycin	0.00	-----	0.00	0.00	-----	0.00	0.67	-----	0.52
Tetracycline	0.00	-----	0.00	0.44	-----	0.43	0.69	-----	0.73
Sulfamethazine	0.00	-----	0.00	0.00	-----	0.00	0.00	-----	0.00
Sulfamethoxazole	1.31	-----	1.22	0.00	-----	1.10	1.42	-----	1.18
Antibacterial compounds									
Triclosan	0.92	-----	0.81	0.38	-----	0.41	1.01	-----	0.95
Amphenicol	-----	0.00	-----	-----	0.00	-----	-----	0.00	-----
Macrolide	-----	0.00	-----	-----	0.00	-----	-----	0.00	-----
ß-Lactam	-----	0.00	-----	-----	0.00	-----	-----	0.00	-----
Chloramphenicol	-----	0.00	-----	-----	0.00	-----	-----	0.00	-----
Gentamicin	-----	0.00	-----	-----	0.00	-----	-----	0.00	-----

1 HPLC=High-performance liquid chromatography.

2 ELISA=Enzyme linked immunosorbent assay. Pesticides were determined by

3 GC=Gas chromatograph-mass spectrometer. Maximum residue limits (ng/g)=MRL streptomycin (500), tetracycline (100), Sulfamethazine (100), sulfamethoxazole (100), triclosan (10), testosterone (0.1), trenbolone (10), estrogen (0.1), 2,4 D (50), DDE/DDT (1000), alachlor (100). DDE=Dichlorodiphenyldichloroethylene, DDT=Dichlorodiphenyltrichloroethane

**Table-5:** Mean concentrations of residues of pesticides, anabolic steroids, antibiotics, and antibacterial compounds (ng/g) in five samples of frozen buffalo meat samples available in local markets in Oman.

Contaminant		Brand				

		Brand 1	Brand 2	Brand 3	Brand 4	Brand 5
Pesticides						
2,4 D	GC	0.00	0.00	0.00	0.00	0.00
	ELISA	17.9	6.79	28.6	31.6	39.7
DDE/DDT	GC	0.00	0.00	0.00	0.00	0.00
	ELISA	0.00	0.00	0.00	0.00	0.00
Alachlor	GC	0.00	0.00	0.00	0.00	0.00
Antibiotics						
Testosterone	HPLC	0.00	0.00	0.00	0.00	0.26
	ELISA	0.00	0.00	0.00	0.00	0.00
Trenbolone	HPLC	ND	0.69	0.68	0.70	ND
	ELISA	0.68	0.86	0.65	0.42	0.53
Estrogen	HPLC	0.62	0.40	1.04	1.12	0.69
Antibiotics						
Streptomycin	HPLC^1^	0.55	0.84	2.01	5.56	3.22
	CHARM	0.00	0.00	0.00	0.00	0.00
	ELISA	0.65	0.77	3.01	4.49	2.99
Tetracycline	HPLC1	0.49	0.19	0.65	0.79	0.95
	CHARM	-----	-----	-----	-----	-----
	ELISA	0.39	0.15	0.51	0.98	1.15
Sulfamethazine	HPLC	0.00	0.00	0.00	0.00	0.00
	ELISA	0.00	0.00	0.00	0.00	0.00
Sulfamethoxazole	HPLC	1.11	1.01	1,16	2.00	1.32
	ELISA	1.17	1.17	1.14	1.19	1.24
Antibacterial compounds						
Amphenicol	CHARM	0.00	0.00	0.00	0.00	0.00
Macrolide	CHARM	0.00	0.00	0.00	0.00	0.00
ß-Lactam	CHARM	0.00	0.00	0.00	0.00	0.00
Chloramphenicol	CHARM	0.00	0.00	0.00	0.00	0.00
Gentamicin	CHARM	0.00	0.00	0.00	0.00	0.00

1 HPLC=High-performance liquid chromatography. ^2^ELISA=Enzyme linked immunosorbent assay. Pesticides were determined by ^3^GC=Gas chromatograph-mass spectrometer. Maximum residue limits (ng/g)=MRL streptomycin (500), tetracycline (100), sulfamethazine (100), sulfamethoxazole (100), triclosan (10), testosterone (0.1), trenbolone (10), estrogen (0.1), 2,4 D (50), DDE/DDT (1000), Alachlor (100). DDE=Dichlorodiphenyldichloroethylene, DDT=Dichlorodiphenyltrichloroethane

**Table-6:** Mean concentrations of residues of pesticides, anabolic steroids, antibiotics, and antibacterial compounds (ng/g) in five samples of beef and buffalo minced meat samples available in local markets in Oman.

Contaminant		Species						

		Beef					Buffalo	
	
		Brand 1	Brand 2	Brand 3	Brand 4	Brand 5	Brand 6	Brand 1
Pesticides								
2,4 D	GC	0.00	0.00	0.00	0.00	0.00	0.00	0.00
	ELISA	25.9	36.0	17.7	15.2	31.0	19.0	20.1
DDE/DDT	GC	-----	0.00	0.00	0.00	0.00	0.00	0.00
	ELISA	0.00	0.00	0.00	0.00	0.00	0.00	0.00
Alachlor	GC	0.00	0.00	0.00	0.00	0.00	0.00	0.00
	ELISA	0.37	0.36	0.14	0.29	0.99	0.25	0.25
Organophosphate	GC	0.00	0.00	0.00	0.00	0.00	0.00	0.00
	ELISA	1.95	1.86	2.00	1.98	1.81	1.94	1.03
Anabolic steroids								
Testosterone	HPLC	0.00	0.00	0.00	0.00	0.00	0.00	0.00
	ELISA	0.00	0.00	0.00	0.00	0.00	0.00	0.00
Trenbolone	HPLC	0.55	0.60	0.49	0.62	0.55	0.59	0.75
	ELISA	0.67	0.71	0.56	1.02	0.99	0.68	0.86
Estrogen	HPLC	1.57	1.59	1.65	1.74	1.89	1.33	0.99
Antibiotics								
Streptomycin	HPLC^1^	0.00	0.00	0.00	0.00	1.94	0.00	0.00
	CHARM	0.00	0.00	0.00	0.00	0.00	0.00	0.00
	ELISA^2^	0.00	0.00	0.00	0.00	2.01	0.00	0.00
Tetracycline	HPLC	0.00	0.19	0.00	0.00	0.00	0.00	0.00
	CHARM	0.00	0.00	0.00	0.00	0.00	0.00	0.00
	ELISA	0.00	0.18	0.00	0.00	0.00	0.00	0.00
Sulfamethazine	HPLC	0.00	0.00	0.00	0.00	0.00	0.00	0.00
	ELISA	0.00	0.00	0.00	0.00	0.00	0.00	0.00
Sulfamethoxazole	HPLC	1.01	1,11	1.13	1.30	1.21	1.15	1.19
	ELISA	1.15	1.17	1.14	1.25	1.07	1.12	1.21
Antibacterial compounds								
Triclosan	HPLC	0.00	0.00	0.00	0.00	0.00	0.00	0.00
	ELISA	0.88	0.94	0.82	0.34	0.48	0.40	0.45
Amphenicol	CHARM	0.00	0.00	0.00	0.00	0.00	0.00	0.00
Macrolide	CHARM	0.00	0.00	0.00	0.00	0.00	0.00	0.00
ß-Lactam	CHARM	0.00	0.00	0.00	0.00	0.00	0.00	0.00
Chloramphenicol	CHARM	0.00	0.00	0.00	0.00	0.00	0.00	0.00
Gentamicin	CHARM	0.00	0.00	0.00	0.00	0.00	0.00	0.00

1 HPLC=High-performance liquid chromatography.

2 ELISA=ELISA=Enzyme linked immunosorbent assay. Maximum residue limits (ng/g)=MRL streptomycin (500), tetracycline (100), sulfamethazine (100), sulfamethoxazole (100), triclosan (10), testosterone (0.1), trenbolone (10), estrogen (0.1), 2,4 D (50), DDE/DDT (1000), alachlor (100). DDE=Dichlorodiphenyldichloroethylene, DDT=Dichlorodiphenyltrichloroethane

**Table-7:** Mean concentrations of residues of pesticides, anabolic steroids, antibiotics, and antibacterial compounds (ng/g) in five samples of sheep minced meat samples available in local markets in Oman.

Contaminant		Brand 1	Brand 2	Brand 3	Brand 4	Brand 5

Pesticides						
2,4 D	GC^3^	-----	-----	-----	-----	-----
	ELISA	14.3	7.79	25.4	6.32	45.9
DDE/DDT	GC	-----	-----	-----	-----	-----
	ELISA	0.00	0.00	0.00	0.00	0.00
Alachlor	GC	-----	-----	-----	-----	-----
	ELISA	1.22	1.09	0.61	0.94	0.76
Organophosphate	GC	-----	-----	-----	-----	-----
	ELISA	1.99	2.05	1.98	2.00	1.99
Anabolic steroids						
Testosterone	HPLC	0.00	0.00	0.00	0.00	0.00
	ELISA	0.00	0.00	0.00	0.00	0.00
Trenbolone	HPLC	0.90	0.87	0.75	1.12	0.67
	ELISA	0.49	0.59	0.29	0.38	0.54
Estrogen	HPLC	1.54	2.19	2.03	1.82	1.72
	ELISA	1.53	1.85	1.90	1.18	1.00
Antibiotics						
Streptomycin	HPLC^1^	5.72	0.00	0.00	2.62	0.53
	CHARM	-----	-----	-----	-----	-----
	ELISA^2^	4.77	0.00	0.00	1.99	0.66
Streptomycin	HPLC	0.00	0.00	0.00	0.00	0.00
	CHARM	-----	-----	-----	-----	-----
	ELISA	0.00	0.00	0.00	0.00	0.00
Sulfamethazine	HPLC	0.98	0.00	0.00	0.32	0.94
	ELISA	1.88	0.00	0.00	0.29	0.84
Sulfamethazine	HPLC	0.00	0.00	0.00	0.00	0.00
	ELISA	1.15	1.23	1.25	1.23	1.12
Antibacterial compounds						
Triclosan	HPLC	0.31	0.52	0.22	0.21	0.42
	ELISA	0.52	0.79	0.24	0.22	0.64
Amphenicol	CHARM	-----	-----	-----	-----	-----
Macrolide	CHARM	-----	-----	-----	-----	-----
ß-Lactam	CHARM	-----	-----	-----	-----	-----
Chloramphenicol	CHARM	-----	-----	-----	-----	-----
Gentamicin	CHARM	-----	-----	-----	-----	-----

1 HPLC=High-performance liquid chromatography.

2 ELISA=Enzyme linked immunosorbent assay. Pesticides were determined by

3 GC=Gas chromatograph-mass spectrometer. Maximum residue limits (ng/g)=MRL streptomycin (500), tetracycline (100), sulfamethazine (100), sulfamethoxazole (100), triclosan (10), testosterone (0.1), trenbolone (10), estrogen (0.1), 2,4 D (50), DDE/DDT (1000), alachlor (100). DDE=Dichlorodiphenyldichloroethylene, DDT=Dichlorodiphenyltrichloroethane

**Table-8:** Mean concentrations of residues of pesticides, anabolic steroids, antibiotics, and antibacterial compounds (ng/g) in five samples of beef sausage samples available in local markets in Oman.

Contaminant	Brand 1			Brand 2			Brand 3		

	Method of analysis								

	HPLC^1^/GC^2^	CHARM	ELISA^32^	HPLC/GC	CHARM	ELISA	HPLC/GC	CHARM	ELISA
Pesticides									
2,4 D	-----	-----	30.3	-----	-----	29.9	-----	-----	31.5
DDE/DDT	-----	-----	-----	-----	-----	-----	-----	-----	-----
Alachlor	-----	-----	0.52	-----	-----	0.36	-----	-----	0.41
Organophosphate	-----	-----	1.97	-----	-----	1.97	-----	-----	1.88
Anabolic steroids									
Testosterone	0.65	-----	0.68	0.71	-----	0.69	0.71	-----	0.73
Trenbolone	0.59	-----	0.79	0.00	-----	0.66	0.00	-----	0.61
Estrogen	1.62	-----	1.72	1.56	-----	1.25	1.79	-----	1.37
Antibiotics									
Streptomycin	0.00	-----	0.00	0.00	-----	0.00	0.00	-----	0.00
Tetracycline	0.16	-----	0.17	0.00	-----	0.00	0.16	-----	0.15
Sulfamethazine	0.00	-----	0.00	0.00	-----	0.00	0.00	-----	0.00
Sulfamethoxazole	0.00	-----	1.06	0.00	-----	1.15	0.00	-----	1.22
Antibacterial compounds									
Triclosan	0.00	-----	0.61	0.54	-----	0.43	0.32	-----	0.58
Amphenicol	-----	-----	-----	-----	-----	----	-----	-----	-----
Macrolide	-----	-----	-----	-----	-----	----	-----	-----	-----
ß-Lactam	-----	-----	-----	-----	-----	----	-----	-----	-----
Chloramphenicol	-----	-----	-----	-----	-----	----	-----	-----	-----
Gentamicin	-----	-----	-----	-----	-----	----	-----	-----	-----

1 HPLC=High performance liquid chromatography; Pesticides residues were determined using GC

2 =Gas chromatography mass spectrometer;

ELISA=Enzyme linked immunosorbent assay. Maximum residue limits (ng/g)=MRL streptomycin (500), tetracycline (100), Sulfamethazine (100), sulfamethoxazole (100), triclosan (10), testosterone (0.1), trenbolone (10), estrogen (0.1), 2,4 D (50), DDE/DDT (1000), Alachlor (100). DDE=Dichlorodiphenyldichloroethylene, DDT=Dichlorodiphenyltrichloroethane

### Pesticide residues

The current results show that meat and meat product samples contained low levels that were below MRLs for pesticides (2,4 D, DDE/DDT, alachlor, organophosphate) (Tables-2-8). In line with the present results, Khalid *et al*. [31] and Ibrahim *et al*. [15] found that pesticide residues in meat samples from ruminants were below the MRLs. The average values of the concentrations (ng/g wet weight) of DDTs in camel, cattle, and sheep muscles were 13.9, 17.9, and 20.3, respectively, were below MRLs [31]. In contrast, residues of pesticides have been found in meat products in different animal species [32]. An LC-MS/MS multi-residue method teamed with a modified quick, easy, cheap, and effective extraction method has been used by many investigators for the investigation of many chemical residues [33-36]. The mean concentrations of the pesticides ranged between 74.7% and 113.5%, for chicken, swine and bovine muscle, and liver tissues. Most of the organochlorine pesticides remained in use by farmers in great amounts as insecticides that act against a wide range of agricultural pests. Most of the pesticides show environmental persistence, which means they can bioaccumulate in animal organs [37]. According to Garcia and Gotah [14], polychlorinated biphenyls (PCBs) are another type of persistent organic pollutants that may contaminate meat products [14]. Although PCBs have been banned in many countries, their residues are still contaminating meat products because of their stability and lipid-soluble properties. Short-term exposures to elevated concentrations of different pesticides may result in illness and death. Whereas chronic exposure to high or low concentrations of pesticides may cause immune dysfunction, endocrine distraction, reproductive and neuro-behavioral syndromes, developing abnormalities, reduced immunity in infants and developmental disorders in children and cancer induction [38]. Organochlorine pesticides can accumulate in meat products and get biomagnified through the food chain, with approximately 97% of the total daily intakes of these compounds in humans are from the diet. Moreover, potential mechanisms of action on humans at toxic doses may involve signs and symptoms of intoxication, excitability, and convulsions. Death may occur between 2 and 12 h after exposure if appropriate treatment is not applied immediately. Community awareness of the negative human health impacts of organochlorine contaminated products has led to strict regulations on their use in many countries. DDT and several other organochlorine compounds, however, are still being unlawfully used for agriculture and animal husbandry in many developing countries, which can cause accumulation of the contaminants in food products, in particular those with excessive fat content such as animal tissues that are responsible for the dietary intakes of most of the organochlorine pesticides [39]. The higher contamination of foods with DDT in developing countries might be due to illegal use of DDT for agriculture productions. Although the levels of pesticide residues in meat products were low, they still may cause harm to human health after long-term consumption.

### Anabolic steroids

Of the anabolic steroids considered (testosterone, trenbolone, and estrogen), estrogen residues in fresh beef (Table-2) and sheep (Table-3) meat samples were beyond the acceptable level by both the HPLC and ELISA techniques. The European Union Commission [40] declared that the MRL of testosterone, trenbolone, and estrogen residues in food should not exceed 0.10, 10.0, and 0.10 ppb, respectively. As shown in Tables-2 and 3, higher concentrations were found in the current study. This study revealed that to remain within acceptable tolerance limits stated above, the use of testosterone, trenbolone, and estrogen in animal husbandry must be controlled. Moreover, the tolerance limit stated by the World Health Organization for the three anabolic steroid residues were exceeded in the present study. The present findings provide a warning about the dangers of using anabolic steroids with animals that are to be used to provide red meat in local markets. Anabolic steroids are classified as growth promoters as they can enhance growth rates and feed conversion ratios in animals along with increased lean bulk and reduced fat mass [13,41]. Anabolic steroid hormones have been used as growth-promoting steroids in farm animals for more than four decades [42]. Estrogen regulates body cell metabolism, and specific features critical in reproduction of the female animal [43]. Estradiol-17 b can be implemented with testosterone, progesterone, or trenbolone acetate to improve weight gain or feed conversion ratio in animals [44]. In fattening livestock, the application of estrogens to enhance body weight gain has been certified in several countries but not in the European Union. However, the presence of anabolic steroid residues in animal tissues might possibly be hazardous to human health due endocrine effects and possible carcinogenic effects. Therefore, the development of multi-concentration analytical techniques has been crucial for the control of possible illegal use of anabolic steroids in animal husbandry.

The mean levels of testosterone, and estrogen residues in two brands of frozen beef and one brand of frozen sheep were above the MRLs (Table-4). Although use of most of anabolic steroids, including estrogen and testosterone is not permitted in most countries producing meat and meat products, illegal use or insufficient withholding periods before slaughter (60-70 days) may lead to unacceptable residues. Therefore, the use of hormones in animal husbandry must be firmly controlled. However, hormones have been frequently used in livestock production. Anabolic residues must be below the MRLs; otherwise, they will be considered as potential risks to human health.

Table-7 shows concentrations of anabolic steroid residues in five brands of minced-sheep meat products available in Omani markets. Among the three anabolic steroids tested, residues of estrogen in sheep minced meat products were above the MRLs. Although, anabolic steroids as growth promotants were banned in many countries, some farmers are still using them to enhance growth of their livestock. The average estrogen residues ranged from 1.54 to 2.19 ppb and 1.00 to 1.90 ppb using HPLC and ELISA techniques, respectively. Minced meat products are considered rich in nutritional values and therefore, they should be safe for human consumption and they should not contain harmful residues that affect human health. However, this study confirmed that the anabolic steroids used in animal production leave residues behind and thus are considered harmful to human health [5]. The European countries prohibited the use of anabolic steroids as growth accelerators in animals while the United States of America allowed the limited use of some anabolic steroids.

The results of anabolic steroids in five brands of buffalo frozen meat samples are presented in Table-5. The average estrogen residues in Brand 1 (0.62 HPLC and 1.52 ELISA), Brand 2 (0.40 HPLC and 1.27 ELISA), Brand 3 (1.04 HPLC and 1.35 ELISA), Brand 4 (1.12 HPLC and 1.07 ELISA), and Brand 5 (0.69 HPLC and 0.47 ELISA) were above the MRLs. As with beef and sheep, illegal anabolic steroids were applied to improve meat production, which may expose humans to excess estrogen and affect health. Therefore, there is a need to regularly screen for anabolic steroids as a meat safety control measure.

Residues of estrogen in six brands of beef and one brand of buffalo minced meat were above MRLs (Table-6). The results of the present study suggest that beef cattle and buffalo were implanted with anabolic steroids within a short time prior slaughter without a suitable withdrawal time. Humans will have high potential exposure to anabolic steroid through the consumption of commercially-available beef and buffalo minced meats in the Omani market with resulting health concerns for consumers. Although, the concentrations of trenbolone are below MRLs, it appears to have extensive illicit use as an anabolic steroid in human body.

Beef sausage preparation from three different meat brands available in Omani markets differed slightly in testosterone, trenbolone, and estrogen levels (Table-8).

### Antibiotics

Residues of antibiotics were low and below the MRLs values established by the EU law of drugs [40]. The acceptable MRL for antibiotic residues as indorsed by the FAO and WHO Committee on Food Additives is 200, 600, and 1200 mg/kg for muscles, liver, and kidney, respectively. Prevalent use of antibiotics in treatment animals without withdrawal periods may lead to accumulation of the drugs in animal tissues [14,45]. The present results indicate that antibiotics have been used at least once during the animal’s lifetime for the treatment of bacterial infections. Antibiotic residues refer to drug prototypes, toxicologically significant metabolites, and drug impurities that accumulate in meat after application [46]. Veterinary drugs belong to several pharmacological classes including antiparasitic, antibacterial, antifungal, anti-inflammatory drugs, and anabolic steroids. Veterinary drugs are widely used for disease treatments and protection of animal health, in addition to other chemical substances such as growth promoters [47-49]. However, the inappropriate use of drugs may result in concentrations in edible portions of animals that can be toxic and hazardous for human health and possibly cause allergic reactions. Small contaminations of antibiotics in animal tissues consumed for long times may cause spread of drug-resistant microorganisms [50,51]. This can contribute to increases in human exposure to antibiotic residues, development of antibiotic-resistant pathogens and increased allergies [14,45,52]. Health hazard anxieties are high regarding possible antibacterial resistance in zoonotic pathogens (*Salmonella* spp. and *Campylobacter* spp.), bacteria (*Escherichia coli* and *Enterococci*), and bacterial pathogens of animals (*Pasteurella* and *Actinobacillus* spp.) [53]. Monitoring of antibiotic residues is very important in controlling the safety of products for human consumption [54]. Associated problems include mutagenicity, nephropathy, immunopathological consequences, toxicity, transfer of antibiotic-resistant bacteria to humans, reproductive disorders, carcinogenicity, hepatotoxicity, bone marrow toxicity, and allergy [5]. Ignoring the instructions on how and when to use antibiotics may lead to antibiotic residues entering animal products [55]. The significance of the chemical contaminants depends on the pharmacodynamics of the chemical and the animal species [56]. According to Eltayb *et al*. [57], many farmers use veterinary drugs for the inhibition and the control of the diseases; but only 5% of animal growers use veterinary drugs to maintain the health status of the livestock.

### Antibacterial compounds

CHARM II was the method used to detect residues of macrolide, ß-lactam, chloramphenicol, and sulfa drugs and gentamicin in red meat products (fresh, frozen, minced, sausages, and burgers). The results showed that 135 samples of red meat products available in local market were free from the above contaminants. Chloramphenicol is an antibiotic product used to treat a number of bacterial infections. The widespread of chloramphenicol in developing countries is due to its low price and ease of manufacture. Although the use of chloramphenicol as a veterinary drug is highly restricted, it has some very important veterinary indications against a variety of Gram-positive and Gram-negative bacteria [58]. The most serious adverse effect associated with chloramphenicol residues in foods is that it may lead to bone marrow toxicity, aplastic anemia, and death [6]. Gentamicin is an aminoglycoside antibiotic comprised a mixture of many gentamicin components. It is used to treat bacterial infections, particularly Gram-negative bacteria infections [59]. Gentamicin is nephrotoxic and ototoxic as well and its poisonousness is continuing to be a major problem in clinical use. Sulfonamide or sulfonamide is the basis of several groups of drugs that are used as synthetic antimicrobial steroids [5]. Beta-lactam constitutes a significant portion of the core structure of many antibiotic groups, the major ones being penicillin, cephalosporins, carbapenems, and monobactams all of which act by preventing bacterial cell wall biosynthesis.

HLPC, ELISA, GC, and CHARM II are reliable analytical methods for residue detection in food products and they have been used by many researchers for similar purposes. For all the methods used in this study, successful recovery tests were conducted. Therefore, the obtained results should be reliable in providing information on the type and quantities of residues of pesticides, anabolic steroids, antibiotics, and antibacterial compounds in meat products available in Omani markets. Some of these residues at certain concentrations are considered to be of high risk to public health. However, due to differences between concentrations of anabolic steroids found in meat products, further investigations are required to determine which specific residues pose the highest risk in meat products.

## Conclusion

The present investigation was carried out to determine levels of chemical residues in meat products available in the Omani market. The results showed that the levels of streptomycin, tetracycline, sulfamethazine, and sulfamethazine residues were low and below the MRL. However, the levels of anabolic steroid residues (testosterone and estrogen) were found in most meat products above the MRLs. Anabolic steroid residues and their metabolites in meat products may have adverse toxic or carcinogenic effects. The levels of antibacterial compounds and pesticides in meat products were below the MRLs, but traces of these chemical compounds may have adverse effects on human health. Long-term human exposure to meat products contaminated with pesticides, veterinary drugs and antibacterial compounds are considered as a health hazard. Moreover, it was found that HPLC, ELISA, GC, and CHARM II methods are appropriate for the detection and quantification of various residues in meat products. Further clinical investigations are necessary to identify residues of highest risk to consumers’ health and wellbeing.

## Authors’ Contributions

IA, ITK, AA, RA, and SK collected data and wrote the first draft of the manuscript. ITK, RA, FM, AH, and KA designed the study. IA, ITK, and AH reviewed and updated the manuscript. All authors read and approved the final manuscript.
